# Comparison of Predictability in Vault Using NK Formula and KS Formula for the Implantable Collamer Lens Surgery

**DOI:** 10.1155/2024/4256371

**Published:** 2024-07-27

**Authors:** Xin Zhong, Yan Li, Yuancun Li, Geng Wang, Yali Du, Mingzhi Zhang

**Affiliations:** Joint Shantou International Eye Center of Shantou University The Chinese University of Hong Kong, Shantou, China

## Abstract

**Background:**

This study aims to investigate the agreement between the NK and KS formulas in predicting the vault after implantation of an EVO-implantable collamer lens (ICL).

**Methods:**

This retrospective study included 106 eyes of 57 patients who underwent ICL-V4c implantation. Preoperative vault prediction was conducted by utilizing the NK and KS formulas, with postoperative measurements by anterior segment optical coherence tomography (AS-OCT) at one month. The analysis focused on the consistency between predicted and achieved vaults, as well as the correlation between the achieved vault and various biometric parameters.

**Results:**

The mean achieved vault was 605.25 ± 212.72 *µ*m, which was significantly smaller than the predicted vaults of 710.08 ± 195.08 and 673.80 ± 212.76 *µ*m, using the NK and KS formulas, respectively (*P* < 0.05). The mean differences between the achieved vault and the predicted vault using the NK formula and KS formula were −104.82 *μ*m (95% LoA: −600.38–391.19 *μ*m) and −68.55 *μ*m (95% LoA: −628.91–491.82 *μ*m), respectively. Anterior chamber depth (ACD), vertical sulcus-to-sulcus (V-STS) diameter, and crystalline lens rise (CLR) were independent factors associated with the achieved vault (*P* < 0.05). The two formulas showed no statistically significant difference in absolute prediction error (APE).

**Conclusion:**

The NK formula exhibited superior consistency and low predictive error compared to the KS formula in the 12.6 mm ICL group. AS-OCT measurements overestimated the predicted ICL vault, especially in the 13.2 mm ICL size selection. Relying solely on the NK or KS formulas for predicting vaults before ICL surgery is not recommended.

## 1. Introduction

The implantable collamer lens (ICL; STAAR Surgical, Monrovia, CA, USA), a posterior chamber phakic intraocular lens, has been reported to have good long-term safety, effectiveness, and predictability [1, 2]. The ICL V4c, invented by Shimizu et al., has a central artificial hole with a 0.36 mm diameter, eliminating preoperative iridotomy and reducing the risk of anterior subcapsular cataract formation [3]. In addition, the presence of the central hole does not significantly affect the visual and refractive outcomes of patients [4].

Long-term follow-up of ICL-V4c implantation indicates that a low vault is associated with a reduced risk of anterior lens opacities [5]. However, oversized ICLs can lead to excessive vaulting (>1000 *µ*m), causing forward displacement of the iris and resulting in acute angle closure and intraocular pressure elevation. An optimal postoperative vault ranges from 250 *µ*m to 750 *µ*m, with consideration for variability due to lighting conditions [6]. The selection criteria for vaulting in low light conditions were due to the lowest vault observed in this state, which increases the risk of contact between the ICL and the lens [7]. Precise ocular biometric measurements and optimal ICL size selection are crucial for successful ICL implantation. The manufacturer-provided online calculation system, which utilizes white-to-white (WTW) distance and anterior chamber depth (ACD), is the most common method for calculating ICL power and size.

Advancements in swept-source anterior segment optical coherence tomography (AS-OCT) have improved the precision of anterior segment biometric measurements, allowing vault prediction via the NK and KS formulas integrated into AS-OCT devices (CASIA2, Tomey, Nagoya, Japan) [8]. The predicted vault using the NK formula was based on anterior chamber width (ACW) and crystalline lens rise (CLR), which represented the distance between nasal to temporal of scleral spurs and vertical dimension to connecting the bilateral angle corners, respectively [9]. The NK formula installed in the AS-OCT shows better accuracy for predicting vault when compared to the STAAR nomogram [9]. Different from the NK formula, the predicted vault using the KS formula was based on the distance of angle to angle (ATA) [10]. The ATA measured by AS-OCT has been reported to be strongly correlated with sulcus-to-sulcus diameter (STS) distance using ultrasound biomicroscopy (UBM), which can represent optimal ICL size selection over traditional methods by using the WTW distance [11]. The selection of an appropriate formula for accurately predicting the vault before ICL surgery is crucial in ensuring patient safety. In addition, it needs further validation for practical use in a clinical setting. In this study, we investigated the agreement of NK and KS formulas based on AS-OCT measurement in predicting the postoperative vault and further identified the biological parameters influencing the accuracy of these two formulas.

## 2. Methods

### 2.1. Subjects

This study recruited 59 patients (106 eyes) who underwent EVO ICL implantation at the Joint Shantou International Eye Center to correct moderate to high myopia and myopic astigmatism. All subjects were informed of the purpose and procedure of the study. The current study was approved by the local clinical research ethics committee (no. EC20221115) and conducted according to the Declaration of Helsinki. Informed consent was obtained from all subjects. Demographic information and medical history were also collected from the subjects.

The inclusion criteria included the following: (1) age between 21 and 45 years old, (2) stable refraction for at least two years (the increase in diopters (D) within two years before surgery was less than 0.50 D per year), (3) diagnosis of myopia with or without astigmatism, (4) ACD ≥2.8 mm, and (5) endothelial cell density ≥2000 cells/mm^2^. The exclusion criteria were as follows: (1) complications occurring during surgery, (2) refractive media opacity severely disturbing vision, and (3) history of ocular diseases other than myopia and astigmatism.

### 2.2. Measurements

Preoperative measurements included uncorrected distance visual acuity (UDVA), corrected distance visual acuity (CDVA), subjective refraction, intraocular pressure (IOP), slit lamp examination, funduscopic examination, and endothelial cell density. A complete auto tonometer was used to measure the IOP. The noncontact specular microscope was used to assess the endothelial cell density. The value of WTW, central corneal thickness (CCT), corneal topography, and pupil diameter was measured by Orbscan IIz Topographer (Bausch and Lomb, Rochester, NY, USA). The UBM (MD-300L, MEDA Co., Ltd, Tianjin, China) was used to measure the sulcus-to-sulcus diameter (STS). The ATA, CLR, ACW, ACD, and ICL vault were obtained by AS-OCT (CASIA2, Tomey, Nagoya, Japan). This equipment automatically calculated the predicted ICL vault by the build-in prediction formulas, including the NK formula (version 3) and the KS formula (version 3) [9, 10]. All patients underwent these measurements under the same indoor illumination conditions before and after surgery.

### 2.3. Lens Size Selection and Power Calculation

The size of the ICL was selected according to the manufacturer based on WTW and ACD. The ICL power was calculated using a modified vertex formula on the manufacturer's online calculator system. Emmetropia was selected as the target refraction for 101 eyes to minimize the preoperative refractive errors. Regarding the highest ICL power of −18.00 D [12], two eyes in our study were undercorrected with residual refractive errors. Before ICL implantation, the spherical refractive errors were −18.5 D and −17.5 D, respectively. To address this issue, spectacles were used after the ICL implantation surgery.

### 2.4. Implantable Collamer Lens Surgical Procedure

Patients received dilating and cycloplegic agents on the day of surgery. Under topical anesthesia, V4c ICL was implanted through a 3-mm clear corneal incision using an injector cartridge (STAAR Surgical AG). The ICL was placed in the posterior chamber and rotated ≤15 degrees using a manipulator. The achieved vault was measured a month postsurgery. All surgeries were performed by the same surgeon (G.W.)

### 2.5. Statistical Analysis

Data normality was assessed using the Shapiro–Wilk test. Differences between predicted and achieved postoperative vaults were evaluated by paired *t*-tests. Spearman's correlation and multivariate linear regression analyses assessed correlations between postoperative vault and biological parameters. Agreement between predicted and achieved vaults was analyzed using Bland–Altman plots with 95% limits of agreement (LoA; mean difference of two methods ± 1.96 standard deviations). Statistical analysis was performed using SPSS (version 22.0), MedCalc (version 20.0.22), and R software (version 4.40). A *P* value of less than 0.05 was considered statistically significant.

## 3. Results

A total of 57 patients (106 eyes) were included in this study (17 men and 40 women). The mean age was 26.65 ± 3.99 years old. There were 58 eyes (54.72%) implanted with a 12.6 mm ICL size and 45 eyes (42.45%) implanted with a 13.2 mm ICL size. Two eyes were implanted with a 13.7 mm ICL size (1.89%), and one eye was implanted with a 12.1 mm ICL size (0.94%). Toric ICLs were implanted in 71 eyes (67.0%) to correct astigmatism of 1 diopter (D) or higher, aiming to rectify its impact. All toric ICLs were implanted within a horizontal range of 0 to 15°.  Table 1 shows the preoperative characteristics of the patients. The mean UDVA was 1.14 ± 0.28 logarithm of the minimum angle of resolution (logMAR), and the mean CDVA was 0.08 ± 0.17 logMAR. No complications were observed after surgery, and no patients required an ICL exchange.

### 3.1. Predicted Vault and Achieved Vault

Table 2 shows the predicted and the achieved postoperative vault of patients. The achieved vaults were significantly different from the predicted vault using the NK formula (*P* < 0.001) and the KS formula (*P* < 0.01) for all patients. In the 12.6 mm ICL group, the achieved vaults were not different from the predicted vault using the NK formula (*P*=0.679) and the KS formula (*P*=0.347). In the 13.2 mm ICL group, the achieved vaults were different from the predicted vault using the NK formula (*P* < 0.001) and the KS formula (*P* < 0.001). Patients were divided into groups based on the CLR, vertical STS, and ACD measurements. The achieved vaults were not significantly different from the predicted vault using the KS formula in patients with CLR ≤0.08 mm, while the achieved vaults were substantially different from the predicted vault using the KS formula in patients with CLR >0.08 mm. However, when patients used the NK formula, there was a significant difference between the predicted vault and the achieved vault at different distances in CLR.

In patients with smaller vertical STS (≤12.5 mm), the achieved vaults were not significantly different from the predicted vault using the NK and KS formulas. In comparison, the achieved vaults were substantially different from the predicted vault using these two formulas in patients with larger vertical STS (>12.5 mm). In patients with different depths of ACD, the achieved vaults were not significantly different from the predicted vault using the KS formula. In contrast, the achieved vaults were substantially different from the predicted vault using the NK formula.

**Figure 1:**
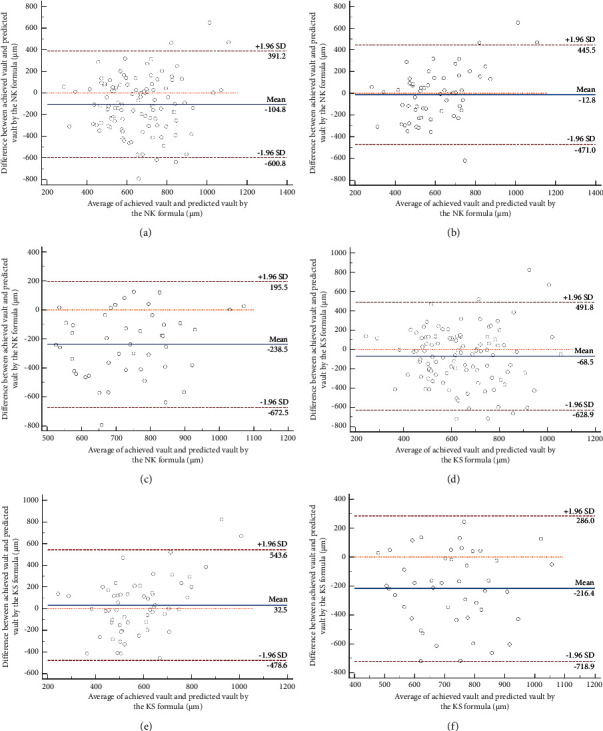
Bland–Altman plot shows the difference between the achieved vault and the predicted vault using the NK formula in all patients (a) and patients with 12.6 mm (b) and 13.2 mm ICL (c). Bland–Altman plot shows the difference between the achieved vault and the predicted vault using the KS formula in all patients (d) and patients with 12.6 mm (e) and 13.2 mm ICL (f).

The agreement between the predicted and achieved postoperative vault was analyzed using the Bland–Altman plots with 95% LoA. The mean differences between the achieved vault and the predicted vault using the NK formula and KS formula were −104.82 *μ*m (95% LoA: −600.38–391.19 *μ*m) and −68.55 *μ*m (95% LoA: −628.91–491.82 *μ*m), respectively. The 95% LoA of the achieved vault and the predicted vault using the NK formula was narrower than those of the achieved vault and the predicted vault using the KS formula. The differences between the achieved vault and the predicted vault for patients implanted with 12.6 mm and 13.2 mm ICLs are shown in the Bland–Altman plots (Figure 1). In the 12.6 mm ICL group, the mean differences between the achieved vault and the predicted vault using the NK formula and KS formula were −12.8 *μ*m (95% LoA: −471.0–445.5 *μ*m) and 32.50 *μ*m (95% LoA: −478.6–543.6 *μ*m), respectively. The 95% LoA of the achieved vault and predicted vault using the NK formula was narrower than that of those obtained with the KS formula in this group. In contrast, in the 13.2 mm ICL group, we observed mean differences between the achieved vault and predicted vault using both formulas as −238.5 *μ*m (95% LoA: −672.5–195.5 *μ*m) for NK formula and −216.4 *μ*m (95% LoA: −718.9–286.0 *μ*m) for KS formula, respectively.

### 3.2. Factors Correlated with Achieved Vault

Table 3 shows the correlations between the achieved vault and the preoperative clinical variables. In the multivariate linear regression analysis, vertical STS (*β* = −0.129; *P*=0.189), CLR (*β* = −0.301; *P*=0.002), and ACD (*β* = 0.309; *P*=0.001) were associated with the achieved vault. In the 12.6 mm ICL group, the ACD (*β* = 0.304; *P*=0.02), CCT (*β* = −0.297; *P*=0.024), WTW (*β* = −0.298; *P*=0.023), CLR (*β* = -0.317; *P*=0.015), horizon STS (*β* = −0.323; *P*=0.013), and vertical STS (*β* = -0.454; *P* < 0.001) were associated with the achieved vault. In the 13.2 mm ICL group, the PD (*β* = 0.324; *P*=0.03) was associated with the achieved vault.

### 3.3. Factors Associated with Predicted Vault

**Figure 2:**
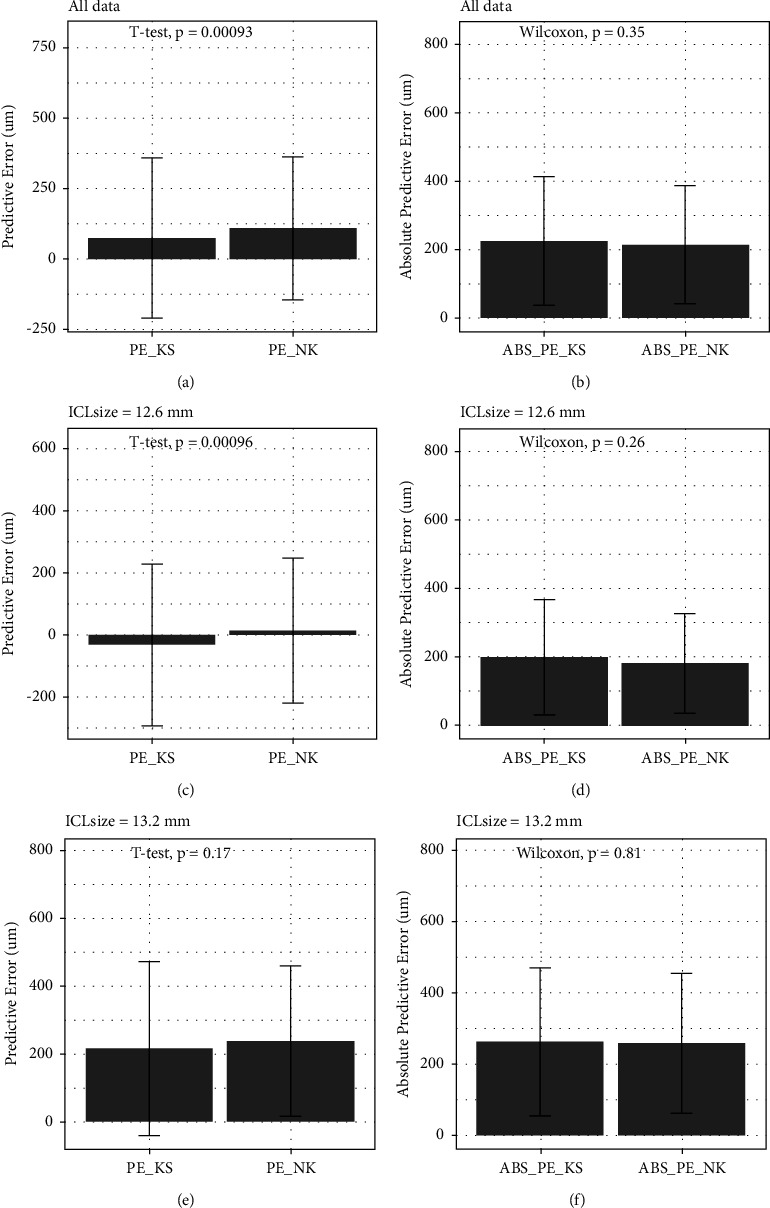
Predicted error and absolutely predicted error in all data (a, b), 12.6 ICL size (c, d), and 13.2 ICL size (e, f). Each box shows the 75th (upper side) and 25th percentiles (lower side).

We also analyze the predicted errors of influence factors using NK and KS formulas. We defined the predicted error (PE) as a different vault between the predicted and postoperatively achieved vault. Absolute predict error (APE) is the absolute vault of PE. As for the NK formula, ICL size (*P* < 0.001) and ATA (*P* < 0.001) were the factors that could bring predicted error to the predicted vault (Supplementary Table 1). In addition to ICL size (*P* < 0.001), ATA (*P* < 0.001), H-STS (*P*=0.004), and CLR (*P*=0.029) were also the factors in predicting error in the KS formula (Supplementary Table 2). The absolute error of the predicted vault size differed significantly between the NK and KS formulas (*P* < 0.001). The box-and-whisker plot for the absolute error (Figure 2) showed that the KS formula had less predictive error in all data, while more predictive error in the 12.6 mm ICL group. However, there was no significant absolute prediction error (APE) difference between the two formulas in Figure 2.

### 3.4. Generalized Estimating Equation (GEE)

In statistical analysis, the generalized estimating equation (GEE) is utilized to estimate the parameters of a generalized linear model, accounting for potential unknown correlations between outcomes. To select an appropriate correlation structure, we evaluated the quasi-likelihood under the independence criterion (QIC) and the quasi-likelihood under independence criterion with corrected sample size (QICC) for a predefined set of model terms, preferring the model with the smallest QIC value. The QICC calculation assumed that the distribution, link function, and working correlation matrix specifications were appropriate for the dataset. Our study compared GEE models with three different correlation structures (exchangeable, AR1, and independence) and calculated their QIC values to identify the best model (Supplementary Table 3). All three models suggested an independent structure, as indicated by their respective smallest QIC values (Supplementary Table 3). Despite incorporating parameters from both eyes of each subject, the databases within this model could be treated as independent.

## 4. Discussion

In this study, we compared the accuracy of the postoperation vault, which was predicted by the NK and KS formulas in AS-OCT, respectively. The NK formula demonstrated a smaller prediction deviation compared to the KS formula, which contrasts with previous research findings [13]. A study found that the mean difference between the achieved and predicted vaults was 64 ± 190 *µ*m (range: −264–742 *µ*m) [14]. Another study reported the mean difference of 19.71 ± 244.08 *μ*m (95% LoA: −498.12–458.69 *μ*m) and 55.79 ± 203.25 *μ*m (95% LoA: −342.56–454.16 *μ*m) using the NK and KS formula, respectively [13]. In contrast, our study observed the mean differences of −104.82 *μ*m (95% LoA: −600.38–391.19 *μ*m) and −68.55 *μ*m (95% LoA: −628.91–491.82 *μ*m) for NK and KS formulas, respectively. These findings indicate an acceptable agreement on the mean value compared to previous studies and highlight a significant range disparity [13, 14]. As shown in Figure 1, the deviation of 12.6 size was obviously narrower than 13.2, representing a more accurate agreement. Previous research has demonstrated that the predicted ICL vault tended to be overestimated using AS-OCT, especially when selecting a 13.2 mm ICL size [15]. Formulas may require adjustment based on ICL size, particularly for the largest and smallest ICL sizes. A comparative analysis of various predictive formulas revealed that both prediction and achieved vaults fell in the targeted 250–750 *µ*m range for ICLs measuring 12.6 and 13.2 mm, which was not observed at the 12.1 and 13.7 mm ICLs [15]. The acceptable range of vaults is relative; a deeper anterior chamber can accommodate a larger vault, while a shallower chamber imposes stricter limitations [16]. Personalized ICL design for each patient is necessary before ICL surgery.

Generally, ICL size is recommended based on WTW and ACD by an online calculator system supported by the manufacturer [8]. However, STAAR's recommended approach only achieved an ideal vault in 69% of patients [17]. Factors such as limbal pigmentation, pannus, or wide greyish limbal edges can impact corneal limbus positioning and ICL sizing calculation [11]. While the haptics of the ICL are typically placed in the ciliary sulcus, determining the appropriate ICL size is ideally based on STS measurements. Studies have shown a limited correlation between STS diameter and WTW measurement [18–21]. UBM-based STS measurements can vary depending on the examiner's experience and skills. Interestingly, our study found no relationship between the horizontal STS and the achieved vault, similar to other research [20, 22]. Instead, vertical STS showed a significant correlation with the achieved vault after surgery (Table 2). Compared to UBM, AS-OCT demonstrated excellent repeatability and accuracy in measuring ocular biological parameters [23].

In our study, the achieved vault significantly differed from the predicted vault using the NK and KS formulas for the 13.2 mm ICL size, but not for the 12.6 mm size. This suggests that larger ICL sizes may have more prediction errors. These results are consistent with previous studies [15, 24]. When a larger ICL size is chosen, there is a higher likelihood of achieving an unpredictable vault. This unpredictability may be attributed to the friction and softness of the ciliary sulcus structure, which can contribute to variations in vault outcomes [20]. The surgical technique is crucial in accurately positioning the ICL footplate, which is essential for the proper vault [22]. The morphology of the ciliary sulcus could potentially be a risk factor for abnormal vault values [25]. Research suggests that the primary factor contributing to this phenomenon is the ciliary sulcus angle (CSA). A smaller ciliary sulcus angle (CSA) hinders adequate footplate contact, leading to an elevated footplate position and increased vault size, while a larger CSA provides less support, reducing the vault [25].

Our finding also demonstrated that ACD, crystalline lens rise (CLR), and H-STS influenced the achieved vault. With the KS formula, CLR, H-STS, ATA, and ICL size were error predictors, while for the NK formula, ATA and ICL size were the error predictors. Adjustments based on these parameters are necessary to minimize prediction errors. Prediction formulas should be modified for different ICL sizes, particularly the larger ones. The distance from the STS line to the CLR apex is relevant to vault size [26]. A larger CLR could occupy more postoperative vault space, leading to a smaller vault. Therefore, the prediction formula should be adjusted for CLR in various ranges. Our results, similar to previous studies [27–29], showed that CLR, STS, and ACD influenced the achieved vault. A greater CLR, STS, and ACD resulted in greater deviations between predicted and achieved vaults. Scotopic and photopic environmental light changes induced a mean change of 59 ± 60 *µ*m in CLR values from mydriasis to miosis [30]. To reduce measurement bias, we measured all subjects under consistent lighting conditions before and after ICL implantation. Ultimately, adjusting vault prediction with personalized parameters may enhance formula accuracy. The focus of further research should be on the development of more efficient algorithms, taking into account all influencing factors, particularly the structure of the ciliary body.

This study has several limitations. The sample size was relatively small. The vault is dynamic and changes with lighting conditions [7], yet we measured it statically. In addition, all patients were Chinese, which might localize biometric parameters and the optimal model [31].

## 5. Conclusions

In summary, the NK formula showed superior consistency and low predictive error compared to the KS formula for the 12.6 mm ICL group. However, both formulas showed suboptimal predictability for larger ICL sizes. AS-OCT measurements tended to overestimate predicted ICL vaults, especially for the 13.2 mm ICL size. Our findings suggest that relying solely on the NK or KS formulas for preoperative vault prediction is not recommended, indicating a need for refinement to enhance predictive accuracy across diverse clinical settings.

## Figures and Tables

**Table 1:** The preoperative characteristics of patients.

Characteristics	Mean ± SD	95% CI (lower, upper)
Age (years)	26.65 ± 3.99	25.88, 27.42
Gender (male/female)	18/41	
UDVA (logMAR)	1.41 ± 0.28	1.35, 1.46
CDVA (logMAR)	0.08 ± 0.17	0.05, 0.11
Manifest spherical refraction (D)	−9.87 ± 3.17	−10.48, −9.26
Manifest cylinder (D)	−1.81 ± 1.24	−2.05, −1.58
IOP (mmHg)	15.50 ± 2.90	14.93, 16.05
Endothelial cell density (cells/mm^2^)	2887.98 ± 257.28	2838.43, 2937.53
Anterior chamber depth (mm)	3.07 ± 0.25	3.03, 3.12
Central corneal thickness (*μ*m)	519.58 ± 40.33	511.81, 527.34
White-to-white distance (mm)	11.67 ± 0.38	11.59, 11.74
Angle-to-angle distance (mm)	11.80 ± 0.40	11.73, 11.88
Crystalline lens rise (mm)	0.07 ± 0.19	0.04, 0.11
Vertical sulcus-to-sulcus diameter (mm)	12.47 ± 0.55	12.36, 12.57
Horizontal sulcus-to-sulcus diameter (mm)	12.04 ± 0.60	11.92, 12.15
Pupil diameter (mm)	4.52 ± 1.09	4.31, 4.73

SD: standard deviation; CI: confidence interval; UDVA: uncorrected distance visual acuity; CDVA: corrected distance visual acuity; *D*: diopter; logMAR: logarithm of the minimal angle of resolution; IOP: intraocular pressure; mm: millimeter; *μ*m: micrometer; mmHg: millimeters of mercury; cells/mm^2^: cells per millimeters squared.

**Table 2:** The achieved postoperative vault and the predicted vault using the NK formula and KS formula.

	Number (%)	Achieved ICL vault (*μ*m)	Predicted ICL vault using the NK formula (*μ*m)^a^	Predicted ICL vault using the KS formula (*μ*m)^a^
Total	106 (100)	605.25 ± 212.72	710.08 ± 195.08^*∗*^	673.80 ± 212.76^*∗*^
ICL size				
12.1 mm	1 (0.94)	574.00	455.00	512.00
12.6 mm	58 (54.72)	595.47 ± 237.88	608.22 ± 147.26	562.97 ± 141.49
13.2 mm	45 (42.45)	616.44 ± 185.16	854.93 ± 156.93^*∗*^	832.89 ± 188.90^*∗*^
13.7 mm	2 (1.89)	653.00 ± 18.38	521.00 ± 41.01	345.00 ± 21.21
Crystalline lens rise				
≤0.08 mm	62 (58.49)	678.68 ± 205.97	776.58 ± 185.52^*∗*^	724.40 ± 205.22
>0.08 mm	44 (41.51)	501.80 ± 177.72	616.36 ± 169.54^*∗*^	602.50 ± 204.70^*∗*^
Vertical sulcus-to-sulcus diameter				
≤12.5 mm	57 (53.77)	619.35 ± 241.74	661.53 ± 170.39	613.30 ± 165.07
>12.5 mm	49 (46.23)	588.86 ± 174.04	766.55 ± 208.14^*∗*^	744.21 ± 240.48^*∗*^
Anterior chamber depth				
≤3.0 mm	44 (41.51)	526.11 ± 185.49	626.09 ± 184.03^*∗*^	604.57 ± 203.80
>3.0 mm	62 (56.60)	661.42 ± 214.26	769.68 ± 181.48^*∗*^	722.94 ± 206.72

mm: millimeter; *μ*m: micrometer. ^a^Compared with the achieved postoperative vault by paired *t*-test. ^*∗*^Significant difference compared with the achieved vault at one month (*P* < 0.05).

**Table 3:** Spearman's correlations between the achieved vault and biological parameters.

	Total		12.6 mm		13.2 mm	
	*r*	*Pvalue*	*r*	*Pvalue*	*r*	*Pvalue*
Age	0.040	0.685	0.043	0.749	0.034	0.825
ACD	0.309	0.001	0.304	0.020	0.290	0.053
CCT	−0.128	0.190	−0.297	0.024	0.137	0.370
WTW	0.041	0.673	−0.298	0.023	0.173	0.256
ATA	−0.015	0.878	−0.127	0.341	−0.012	0.939
CLR	−0.301	0.002	−0.317	0.015	−0.279	0.064
V- STS	−0.129	0.189	−0.454	<0.001	−0.116	0.446
H-STS	−0.087	0.377	−0.323	0.013	−0.092	0.439
PD	0.180	0.065	0.033	0.808	0.324	0.030
ICL size	0.102	0.301	—	—	—	—

ACD: anterior chamber depth; CCT: central corneal thickness; WTW: white to white; ATA: angle to angle; CLR: crystalline lens rise; V-STS: vertical sulcus-to- sulcus; H-STS: horizontal sulcus-to-sulcus; PD: pupil diameter.

## Data Availability

All data generated or analyzed during this study are included in this article.

## Abbreviations

ICL: Implantable collamer lens
WTW: White to white
ATA: Angle to angle
ACD: Anterior chamber depth
STS: Sulcus-to-sulcus diameter
AS-OCT: Anterior segment optical coherence tomography
D: Diopter
UDVA: Uncorrected distance visual acuity
CDVA: Corrected distance visual acuity
IOP: Intraocular pressure
CCT: Central corneal thickness
CLR: Crystalline lens rise
ACW: Anterior chamber width
logMAR: Logarithm of the minimal angle of resolution
SD: Standard deviation
CI: Confidence interval.

## Supplementary Materials

Supplementary Materials Supplemental Table 1 and Supplemental Table 2 show the logistic regression analysis for the predicted error event risk factors of the NK formula and that of the KS formula, respectively. Supplemental Table 3 shows the GEE model with three different correlation structures.
