# Supplementary material for: Comparison of Predictability in Vault Using NK Formula and KS Formula for the Implantable Collamer Lens Surgery
Source: J Ophthalmol. 2024 Jul 27;2024:4256371. doi: 10.1155/2024/4256371 (PMC11300086; doi:10.1155/2024/4256371)
Supplement: Supplementary Materials — Supplemental Table 1 and Supplemental Table 2 show the logistic regression analysis for the predicted error event risk factors of the NK formula and that of the KS formula, respectively. Supplemental Table 3 shows the GEE model with three different correlation structures. [file 4256371.f1.pdf]

Supplemental table 1 Logistic regression analysis for the predict error event risk factors of NK formula

Name	Desc	Value	Coefficient (univariable)	Coefficient (multivariable)	Coefficient (final)
WTW	[11,13]	11.7 ± 0.4	153.45 (16.67 to 290.22, *p*=.028)	49.53 (-132.78 to 231.85, *p*=.591)	
ATA	[10.9,12.8]	11.8 ± 0.4	-31.63 (-162.32 to 99.06, *p*=.632)	-335.34 (-490.81 to -179.88, *p*<.001)	-312.21 (-445.75 to -178.67, ***p*<.001**)
CLR	[-0.3,1.3]	0.1 ± 0.2	-75.48 (-336.31 to 185.36, *p*=.567)	58.58 (-198.78 to 315.94, *p*=.652)	
ACD	[2.6,4.1]	3.1 ± 0.2	22.05 (-187.85 to 231.94, *p*=.835)	-155.33 (-373.99 to 63.34, *p*=.162)	-156.75 (-339.24 to 25.75, *p*=.091)
H-STS	[10.2,13.4]	12.0 ± 0.6	125.78 (45.62 to 205.95, *p*=.002)	70.49 (-26.85 to 167.82, *p*=.154)	69.21 (-22.99 to 161.40, *p*=.140)
ICLsize	[12.1,13.7]	12.9 ± 0.3	371.83 (226.97 to 516.69, *p*<.001)	513.73 (305.44 to 722.02, *p*<.001)	543.21 (356.41 to 730.01, ***p*<.001**)
Age	[21,38]	26.7 ± 4.0	3.15 (-9.19 to 15.49, *p*=.613)	1.22 (-9.51 to 11.95, *p*=.822)	
Gender	Female (N=74)	131.9 ± 256.0			
	Male (N=32)	58.1 ± 243.8	-73.77 (-180.12 to 32.58, *p*=.172)	-18.08 (-113.21 to 77.05, *p*=.707)	

WTW = white to white; ATA = angle to angle; CLR = crystalline lens rise; ACD = anterior chamber depth; H-STS = horizontal sulcus-to-sulcus.

Supplemental table 2 Logistic regression analysis for the predict error event risk factors of KS formula

Name	Desc	Value	Coefficient (univariable)	Coefficient (multivariable)	Coefficient (final)
WTW	[11,13]	11.7 ± 0.4	136.71 (-18.38 to 291.80, *p*=.083)	43.43 (-142.44 to 229.29, *p*=.644)	
ATA	[10.9,12.8]	11.8 ± 0.4	-87.36 (-233.39 to 58.68, *p*=.238)	-513.48 (-671.97 to -354.99, *p*<.001)	-500.89 (-650.16 to -351.62, ***p*<.001**)
CLR	[-0.3,1.3]	0.1 ± 0.2	57.79 (-235.60 to 351.18, *p*=.697)	302.43 (40.07 to 564.79, *p*=.024)	277.14 (29.18 to 525.11, ***p*=.029**)
ACD	[2.6,4.1]	3.1 ± 0.2	-55.35 (-291.04 to 180.34, *p*=.642)	-171.40 (-394.31 to 51.52, *p*=.130)	-177.68 (-376.31 to 20.96, *p*=.079)
H-STS	[10.2,13.4]	12.0 ± 0.6	154.59 (65.34 to 243.85, *p*<.001)	143.06 (43.83 to 242.29, *p*=.005)	142.35 (46.21 to 238.50, ***p*=.004**)
ICLsize	[12.1,13.7]	12.9 ± 0.3	401.06 (236.64 to 565.48, *p*<.001)	576.07 (363.73 to 788.41, *p*<.001)	614.25 (422.38 to 806.12, ***p*<.001**)
Age	[21,38]	26.7 ± 4.0	4.65 (-9.21 to 18.50, *p*=.507)	0.33 (-10.61 to 11.27, *p*=.953)	
Gender	Female (N=74)	103.9 ± 288.5			
	Male (N=32)	9.8 ± 269.5	-94.11 (-213.31 to 25.09, p=.120)	-45.58 (-142.57 to 51.40, p=.353)	

WTW = white to white; ATA = angle to angle; CLR = crystalline lens rise; ACD = anterior chamber depth; H-STS = horizontal sulcus-to-sulcus.

Supplemental Table 3 GEE model with three different correlation structures

Y	structure	QIC	QICu	Quasi Lik	CIC	params	QICC
post_vault_1month	exchangeable	4362141.365	4362140.596	-2181066.298	4.38481273	4	4362142.59
post_vault_1month	ar1	4362141.365	4362140.596	-2181066.298	4.38481273	4	4362142.59
post_vault_1month	independence	**4043349.724**	4043346.97	-2021669.485	5.37671268	4	**4043350.52**
PE_KS	exchangeable	4938859.454	4938856.278	-2469423.139	6.58792036	5	4938861.2
PE_KS	ar1	4938859.454	4938856.278	-2469423.139	6.58792036	5	4938861.2
PE_KS	independence	**4542022.924**	4542018.475	-2271004.238	7.22436624	5	**4542024.15**
PE_NK	exchangeable	4313584.358	4313581.19	-2156786.595	5.58376908	4	4313585.58
PE_NK	ar1	4313584.358	4313581.19	-2156786.595	5.58376908	4	4313585.58
PE_NK	independence	**4309100.608**	4309097.063	-2154544.532	5.77232091	4	**4309101.41**

QIC: Quasi Likelihood under Independence Model Criterion; Quasi Lik: Link Function; QICu: Corrected Quasi Likelihood under Independence; Model Criterion; Quasi Lik: Quasi Likelihood; CIC: Correlated Independence Model Criterion; params: Number of parameters; QICC: Corrected Quasi Likelihood under Independence Model Criterion.

Supplementary statement:

1.exchangeable: Assumes that the correlation between all observations is the same.

2.ar1: Autoregressive structure, assumes correlation decreases over time.

3.independence: Assumes all observations are independent.

In choosing between three models, the model with the smallest QIC criterion measure is preferred.
